# Death of Professor Wagner

**Published:** 1841-08

**Authors:** 


### DEATH OF PROFESSOR WAGNER.

Having corresponded occasionally with Prof. Wagner, of the South Carolina College, for the last fifteen years, it was with more than ordinary regret, that we lately heard of his decease. The particulars have not yet reached us. His successor, we understand, is to be the learned and able Dr. Geddings, who will be transferred from the chair of Pathological Anatomy to that of Surgery. D.

